# Object Detection in Multispectral Remote Sensing Images Based on Cross-Modal Cross-Attention

**DOI:** 10.3390/s24134098

**Published:** 2024-06-24

**Authors:** Pujie Zhao, Xia Ye, Ziang Du

**Affiliations:** Xi’an Research Institute of High-Tech, Xi’an 710025, China; pujie_zhao@outlook.com (P.Z.); dza520@outlook.com (Z.D.)

**Keywords:** remote sensing, multi-spectral, cross-modal information fusion, information enhancement

## Abstract

In complex environments a single visible image is not good enough to perceive the environment, this paper proposes a novel dual-stream real-time detector designed for target detection in extreme environments such as nighttime and fog, which is able to efficiently utilise both visible and infrared images to achieve Fast All-Weatherenvironment sensing (FAWDet). Firstly, in order to allow the network to process information from different modalities simultaneously, this paper expands the state-of-the-art end-to-end detector YOLOv8, the backbone is expanded in parallel as a dual stream. Then, for purpose of avoid information loss in the process of network deepening, a cross-modal feature enhancement module is designed in this study, which enhances each modal feature by cross-modal attention mechanisms, thus effectively avoiding information loss and improving the detection capability of small targets. In addition, for the significant differences between modal features, this paper proposes a three-stage fusion strategy to optimise the feature integration through the fusion of spatial, channel and overall dimensions. It is worth mentioning that the cross-modal feature fusion module adopts an end-to-end training approach. Extensive experiments on two datasets validate that the proposed method achieves state-of-the-art performance in detecting small targets. The cross-modal real-time detector in this study not only demonstrates excellent stability and robust detection performance, but also provides a new solution for target detection techniques in extreme environments.

## 1. Introduction

Object detection based on deep learning is one of the key techniques in the cross-application of machine vision and remote sensing technology [1,2]. Object detection in remote sensing images is a technique that uses images acquired from satellites or drones, etc., to identify, classify and monitor features on the Earth’s surface. These techniques have a wide range of applications in many fields such as military, agriculture, geological exploration, urban planning, and environmental monitoring [3].

Traditional target detection techniques [4,5,6], which mainly use the sliding window on image method, first identify the candidate region on the image, extract the relevant features and classify them using support vector machine. As traditional target detection techniques have high computational complexity, low adaptability and other problems, with the development of deep learning, deep learning based target detection algorithms surpass these traditional detection methods, deep learning based target detection algorithms mainly deal with natural images, which can be divided into two-stage detection algorithms as well as one- stage detection algorithms [7,8], and the two- stage detectors mainly include the Region-based Convolutional Neural Networks (RCNN) family [9,10,11,12], which divides the detection algorithm into two stages: localisation and recognition, and one-stage detection algorithms such as Single Shot MultiBox Detector (SSD) [13], RetinaNet [14], and You Only Look Once (YOLO) series algorithms [15,16,17,18], etc. One-stage detectors use regression to achieve target recognition and localisation, which reduces the region proposing step compared to two-stage detectors, and has a faster speed while detecting with high accuracy. However, these methods are designed for a single modality, and RGB images are susceptible to harsh environments such as low-light scenes or foggy days, for example, leading to poor detection by these target detection algorithms.

However, this drawback can be overcome by introducing additional target information in the imaging mode [19,20]. Considering the robustness of the IR camera to illumination and weather changes, we try to additionally introduce thermal infrared (IR) spectroscopy. IR images measure the temperature of the detected target, thus avoiding the effects of low illumination scenarios and foggy weather scenarios on target detection accuracy [21,22]. When the target is in a scene with insufficient visible light, the target features in the RGB image cannot be extracted, and the corresponding IR image can provide effective information about the object. Zhang et al. [23] designed QFDet to detect small figures in aerial imagery more efficiently by utilising the feature information from both RGB images and thermal IR. An et al. [24] proposed ECISNet that improves the detection accuracy by enhancing the feature representation capability between RGB and thermal infrared modalities. Fusing the complementary modalities of RGB and thermal infrared can further improve the perceivability and robustness in target detection algorithms. Meanwhile, some challenging multispectral datasets, e.g., DroneVehicle [25], VEDAI [26], LLVIP [27], etc., continue to promote the development of multispectral target detection.

In order to better solve the problem of environmental perception in complex environments, this paper proposes a fast all-weather detection algorithm that can simultaneously process complementary visible image information and infrared image information to achieve accurate environmental perception. Specifically, this paper has the following contributions:

1. A dual-stream real-time detector is proposed to perform target detection using both visible and infrared images with stable detection performance in extreme environments such as night and fog.
2. The process of network deepening inevitably brings information loss. In this paper, the features of each modality are filtered and enhanced by cross-modal attention, avoiding the information loss in the process of network deepening, and improving the detection effect of the detector on weak targets.
3. The features of different modalities often possess large differences, and one-time fusion does not mix them well. In this paper, a three-stage fusion strategy is designed to fuse features of different modalities from three different perspectives: spatial, channel, and overall. It is worth noting that the cross-modal feature fusion module is end-to-end during training.
4. Extensive experiments on two datasets show that the method in this paper achieves SOTA performance in the detection of remotely sensed objects.

**Figure 1:**
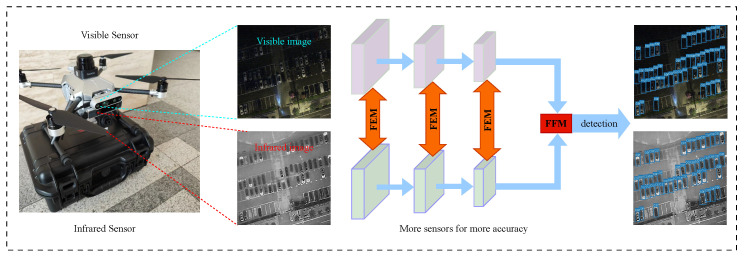
The overall flow of the algorithm in this paper. Where FEM and FFM are the feature enhancement and feature fusion modules proposed in this paper.

Figure 1 shows the overall architecture of the algorithm in this paper. This paper is organized as follows. In Section 2, we introduce the related work. Section 3 presents our method. Section 4 contains experiments. Finally, we conclude in Section 5.

## 2. Related Work

### 2.1. Single Source Remote Sensing Object Detection

Traditional object detection algorithms rely on manual feature extraction, these algorithms are limited in terms of detection efficiency, detection accuracy, and equipment deployment, making them unsuitable for remote sensing equipment cross-application. Most of the deep learning based object detection algorithms use DCNN, for processing remote sensing images, Jiang et al. [28] proposed an optimised deep neural network, where a dual feature map extraction strategy and interleaved localisation strategy were used to optimise the detection of small or narrow rectangular objects. Haroon et al. [29] proposed an adaptive single-pass depth multiscale target detection framework for detecting objects of multiple sizes and different classes from remotely sensed images. Gao et al. [30] proposed a two-stage model for vehicle detection using Fully Convolutional One-Stage Object Detection (FCOS), which is designed with a two-stage positive and negative sample mechanism and a two-step classification model. With the development of regression-based target detection technology, the YOLO series of algorithms has a wide range of applications [31], and the direct cross-application of the original YOLO series of algorithms with remote sensing technology will have low detection accuracy and large model size, so the aerial target detection network based on the YOLO architecture is constantly proposed. Ma et al. [32] proposed Light-YOLOv4 to target the problem of object detection for edge-oriented devices.Light-YOLOv4 performs a series of sparse training, pruning, knowledge distillation, and quantisation operations, which makes Light-YOLOv4 more suitable to be deployed in remote sensing platforms. Liu et al. [33] proposed CCH-YOLOX to solve the aerial images’ problems caused by dense object distribution and scale variations. Deng et al. [34] proposed LAI-YOLOv5s to improve the detection efficiency by combining Deep Feature Map Cross Path Fusion Network for feature fusion and VoVNet module for enhanced feature extraction in the architecture of YOLOv5. Zhang et al. [35] proposed Vit-YOLO, which enhances the detection efficiency by integrating the multi-head self-attention block and BiFPN modules to enhance the detection of small objects. Hui et al. [36] proposed DSAA-YOLO, which enriches the dataset by proposing Super Resolution Data Augment (SRDA) data enhancement strategy to maintain the data quality while enriching the dataset, designing Dense Residual-based Super-Resolution module (DRSR) and Information Alignment Feature Enhancement Module (IAFE) modules to extract the original features of the object of the remotely sensed image in a higher quality, and finally designing the Multi-Object Golf Dynamic Anchor (MGDA) strategy to enhance the effective target feature extraction and generates more accurate bounding boxes, which effectively improves the detection accuracy. It can be seen that the targeted network design effectively improves the target detection efficiency under the condition of sufficient visible light.

### 2.2. Multimodal Remote Sensing Object Detection

The performance of object detection in remote sensing images can be further improved by combining multimodal technology with remote sensing technology. Fusion of multiple modal information in remote sensing images is the core problem of target detection in multispectral remote sensing images, and multispectral fusion methods have been categorised into three forms based on the different stages of fusion [37], i.e., pixel-level fusion, feature-level fusion and decision-level fusion. Pixel-level fusion fuses different modalities at the primary stage, which has a low fusion cost but is sensitive to noise and may not be able to effectively utilise the high-level features and semantic information of the remote sensing image by fusing only at the pixel level. Decision-level fusion fuses the detection results at the final stage, which can utilise the final detection results of each modality and effectively reduce the influence of low-level noise on the final detection results, but this fusion method requires effective decision rules to be formulated in advance and occupies a large amount of hardware resources due to the repeated computation on different modal branches. Aerial image detection networks based on cross-modal fusion mainly use feature-level fusion because feature-level fusion can achieve a better balance between preserving detailed information and providing advanced semantic information, and it is a more applicable method in multimodal remote sensing image fusion.Feature-level fusion firstly inputs the different modal images into parallel branches, which independently extract features from the different modalities, and then, through attention or tandem operations to combine these features. Some works have conducted in-depth studies on RGB-IR multimodal object detection, Sun et al. [25] used feature-level fusion and decision-level fusion in their study, they constructed a UA-CMDet that reduces the detection bias caused by high-uncertainty targets by fusing the information from both modalities of the visual and infrared images and quantifying the uncertainty of the different targets using illumination estimation, thus Vehicle detection in extreme scenarios is achieved. Fang et al. [38] proposed an effective cross-modal feature fusion method based on the self-attention mechanism, which improves the performance of multispectral target detection in remote sensing images by making full use of different modal information. Bao et al. [39] proposed Dual-YOLO, which improves the performance of multi-spectral target detection in remote sensing images by designing Attention Fusion Modul and Fusion Shuffle Module to efficiently process and integrate features from both image types, introducing Fusion Loss to accelerate network convergence during training, by optimising the integration of infrared and visible features. Existing feature-level fusion methods do not deeply explore the characteristics of the information between different modes, and the backbone network does not extract the features well; this study starts from the correlation and differences between modes, and designs a feature enhancement module to enhance the features of different modes, and a feature fusion module to fuse the features of different modes, and selects the state-of-the-art single-stream detection algorithm to expand it, achieves state-of-the-art multi-spectral detection performance.

## 3. Methodology

### 3.1. Algorithm Overview

Yolo algorithm has been widely used due to its high detection accuracy with very fast detection speed. In this paper, YOLOv8 with the best performance is selected as the baseline for expansion. The overall structure of YOLOv8 consists of backbone, neck and detect head, where the backbone part is mainly used for feature extraction, which mainly consists of the CBS module, the C2f module and the SPPF module, the CBS module performs convolutional operations on the input information, followed by batch normalisation, and finally activates the information streams using SiLU in order to obtain the output results. The Ncek part is inspired by PANet [40] and adopts the PAN-FPN structure, the neck part is used to fuse the features extracted from the backbone at different scales, and the detect head is used to process the fused features to get the final detection results.

**Figure 2:**
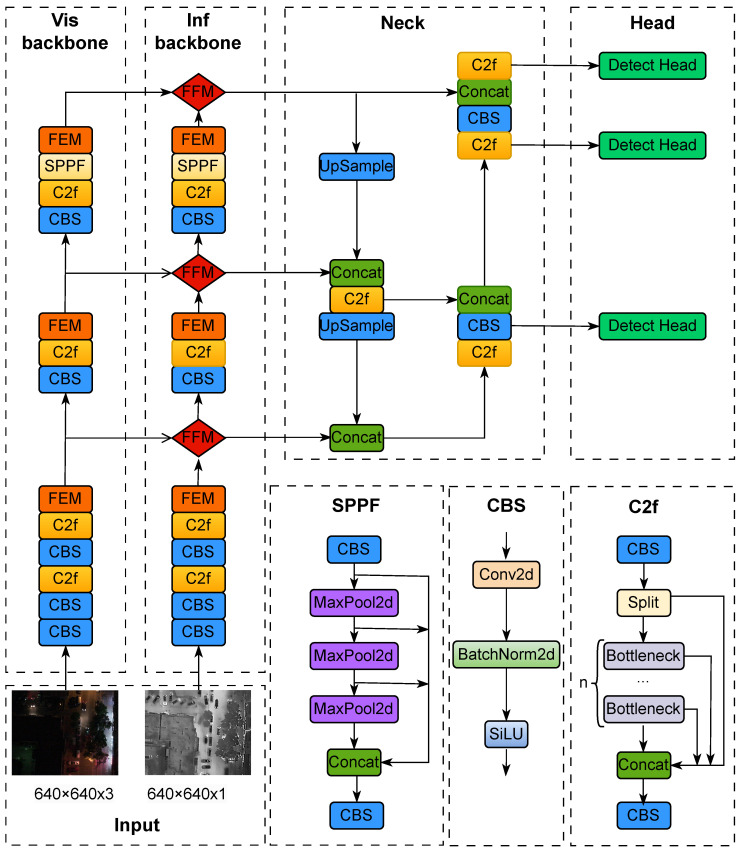
Overall network structure. FEM denotes feature enhancement module and FEM denotes feature fusion module. CBS, C2f, SPPF are single modal feature processing modules.

In this process, a backbone can only extract the features of one modality, but cannot take care of the features of another modality. In order to allow the detector to process the features of different modalities at the same time, early researchers tried to fuse the images of different modalities and then feed them into the detector, which does not take advantage of the complementary modalities very well. Therefore, this paper extends the backbone part to develop a feature extraction backbone for dual streams, and the overall structure is shown in Figure 2. Firstly, we connect two identical backbones in parallel. Then, Cross-modal feature enhancement module (FEM) is embedded between the same layers of different backbones for enhancing the features of different modalities. Finally, Cross-modal feature fusion module (FFM) module is designed to fuse the features coming from the same stages of different backbones. After feature extraction by the dual-stream backbone, the enhanced and fused features are fed into a neck network to fuse the features at different scales, and finally the fused features are fed into the detect head for regression prediction to obtain the final detection results.

### 3.2. Single Module Information Processing Module

The unimodal information processing module aims to efficiently process visible modal features along with infrared modal features, specifically using the C2f module which uses gradient shunt connections to enrich the information flow of the network. The feature maps are pooled using the SPPF module to achieve adaptively sized outputs.

#### 3.2.1. C2f Module

The C2f module has a key role in feature extraction and information flow optimisation, and the C2f module is constructed and designed based on the improvement of the Cross Stage Partial (CSP) [16] structure.The C2f module not only improves the efficiency of feature utilisation, but also increases the network’s ability to process high-dimensional information, while maintaining the lightweight nature of the model. Specifically, the C2f module can be defined as:

(1) $$C2f\left(x\right)=Conv_{2}\left(Concat\left(split_{1}\left(x\right),Bottleneck\left(split_{2}\left(x\right)\right)\right)\right)$$

The input feature *x* is first passed through the convolutional layer $Conv_{1}$, which divides the output into two parts:

(2) $$split_{1}\left(x\right),split_{2}\left(x\right)=split\left(Conv_{1}\left(x\right)\right)$$

where $split_{1}\left(x\right)$ and $split_{2}\left(x\right)$ denote split the output of $Conv_{1}$ into two parts along the channel dimension, following $split_{2}\left(x\right)$ is fed into a series of Bottleneck layers, each is with batch normalization:

(3) $$Bottleneck\left(y\right)=BN\left(Conv\left(y\right)\right)$$

where *y* denote the input feature of Bottleneck, BN denote batch normalization, Conv denote convolution with kernel is 3. The output of each Bottleneck is summed up, the features are then merged with $split_{1}\left(x\right)$ in the channel dimension, Concat denote feature merging along the channel, the result of the final feature merge is processed by the convolutional layer $Conv_{2}$, reducing feature dimensions using convolution with a convolution kernel of 1.

#### 3.2.2. SPPF Module

SPPF is optimised for spatial pyramid pooling (SPP) [41] to extract multi-scale spatial features from the input feature maps while maintaining high computational efficiency.SPPF first passes the input feature maps through a convolutional layer to reduce the dimensionality of the data while keeping the spatial dimensionality of the feature maps constant:

(4) $$x^{\prime }=Conv\left(x\right)$$

where $x^{\prime }$ denote convolution with kernel 1. Next, a maximum pooling operation is performed on the feature maps that have been processed by the first convolutional layer, with the aim of enhancing the model’s ability to perceive features at different scales in the input data without changing the spatial dimensions of the feature maps:

(5) $$y_{1}=Maxpool\left(x^{\prime }\right)$$

(6) $$y_{2}=Maxpool\left(y_{1}\right)$$

(7) $$y_{3}=Maxpool\left(y_{2}\right)$$

where $Maxpool\left(\cdot \right)$ denote maximum pooling. Immediately after that, the input feature map *x* is spliced with the three pooled feature maps $y_{1},y_{2},y_{3}$ in the channel dimension. Finally, the channel dimensions of the feature map are adjusted by another convolutional layer with a convolutional kernel of 1. The output is also obtained by fusing the features at each scale *z*:

(8) $$z=Conv\left(Concat\left(x^{\prime },y_{1},y_{2},y_{3}\right)\right)$$

### 3.3. Cross-Modal Information Processing Module

The cross-modal information processing module aims to process visible modal features and infrared modal features at the same time, and we designed FEM and FFM to reduce the loss of effective feature information and improve the robustness of the model.

#### 3.3.1. Cross-Modal Feature Enhancement Module

**Figure 3:**
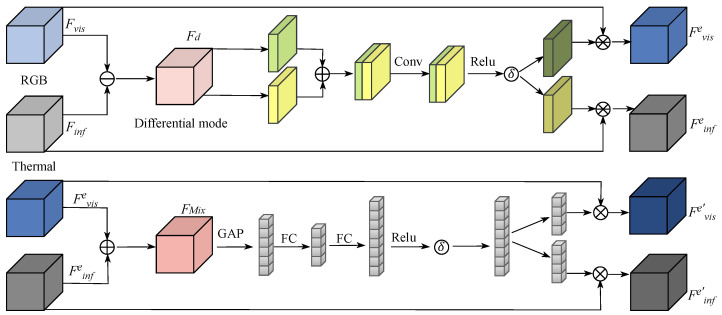
Overall FEM structure. Mixed attention is used to enhance the features of different modalities.

The features of different modalities can be divided into differential mode part and common mode part, during the forward propagation of multiple modalities, the loss of information in the common mode part of one modality can be supplemented by the information of another modality, but the loss of differential mode information is fatal. Therefore, in this paper, a cross-modal feature enhancement module is designed to enhance the differential mode portion of different modal features to combat potential feature loss during network deepening. Figure 3 presents the overall structure of the cross-modal feature enhancement module in this paper. Firstly, the differential mode part of infrared and visible modal features is extracted:

(9) $$F_{d}=\left|F_{vis}-F_{inf}\right|$$

where $F_{d}$ denotes the differential mode part of the dissimilar modal features, $F_{vis}$ denotes visible images features, $F_{inf}$ denotes infrared images features, $\left|\cdot \right|$ denotes absolute value. Next, the information about the spatial distribution of the differential mode part of the features in the visible and infrared features is estimated:

(10) $$M=\sigma \left(ReLU\left(Conv\left(Concat\left(Avepool\left(F_{d}\right),Maxpool\left(F_{d}\right)\right)\right)\right)\right)$$

where *M* denotes the information about the spatial distribution of the differential mode features among the different modal features. σ denote Sigmoid function. ReLU denotes the ReLu activation function. Conv denotes a convolutional layer with a convolutional kernel of 3, step size is set to 1 and padding is set to 1, number of input channels is 2, number of output channels is 1, is used to learn information about the spatial distribution of differential mode features. Concat denote connections along the corridor. Avepool denotes the average pooling along the channel. Maxpool denotes the maximum pooling along the channel, is used for initial feature extraction of differential mode features. Then, using the information of the spatial distribution of the differential mode features of the visible and infrared modes, the features of the different modes are spatially augmented to reinforce the importance of the differential mode features among the different modal features:

(11) $$F_{vis}^{e},F_{inf}^{e}=F_{vis}⊛M,F_{inf}⊛M$$

where $F_{vis}^{e}$ and $F_{inf}^{e}$ denote the visible and infrared features after feature enhancement. ⊛ denotes multiplication of corresponding elements in space. After the spatial enhancement, it is immediately followed by the on-channel enhancement, unlike the spatial enhancement, the channel enhancement learns the channel distribution vectors of the differential mode features in the dissimilar mode features:

(12) W=σReLUFCReLUFCGAPConcatFvise,Finfe

where *W* denote vector representing the channel distribution of differential mode features in different modal features. FC denote the fully connected layer. GAP indicates global average pooling. In this process, the spatially augmented dissimilar modal features are first blended, then the blended feature vectors are compressed into one-dimensional vectors using global average pooling, followed by squeezing and excitation of the vectors using two fully-connected layers, where the number of channels is first squeezed to 1/16 of the input, then enlarged by a factor of 8. Finally, a Sigmoid function is used to constrain the values of the channel vectors to be between 0 and 1. After obtaining the channel distribution vectors, the features of the dissimilar modes are further enhanced to obtain the final output:

(13) $$F_{vis}^{e^{\prime }},F_{inf}^{e^{\prime }}=F_{vis}^{e}⊗W,F_{inf}^{e}⊗W$$

where $F_{vis}^{e^{\prime }}$ and $F_{inf}^{e^{\prime }}$ denote enhanced visible features and infrared features. ⊗ denotes the element-by-element multiplication in the channel dimension.

#### 3.3.2. Cross-Modal Feature Fusion Module

**Figure 4:**
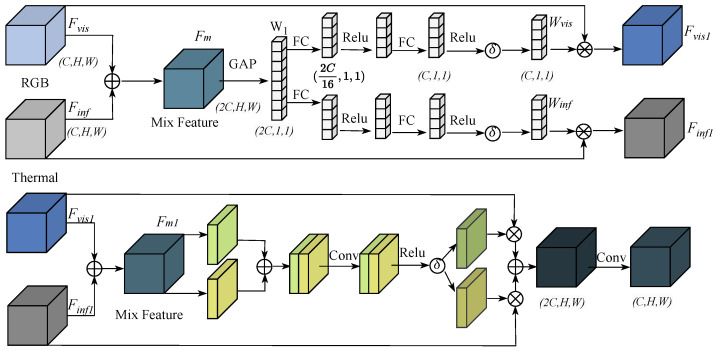
Overall FFM structure.

Features from dissimilar modalities possess different characteristics, and simple summation or connection on channels cannot well fuse features from different modalities. Therefore, in this paper, the Cross-modal feature fusion module (FFM) module is designed to fuse the features extracted from different backbones in different phases, and Figure 4 shows the overall architecture of the FFM module. Firstly, the initial fusion of dissimilar modal features is performed by splicing on the channels. Then the initial fused features are compressed into vectors using a global average pooling layer, and the process can be formulated as:

(14) $$W_{1}=GAP\left(Concat\left(F_{\mathrm{vis}\;},F_{inf}\right)\right)$$

where $W_{1}$ denotes the vector obtained by compressing the features after initial fusion. Next, the vectors are processed using two different squeeze-excitation branches to obtain the channel weights for the fusion of visible and infrared features:

(15) Wvis,Winf=σReLUFCReLUFCW1,σReLUFCReLUFCW1

where $W_{vis}$ and $W_{inf}$ denote the channel weight vectors for visible and infrared feature fusion, respectively, and it is worth noting that the weights of the two squeeze-excitation branches are not shared. Then, the first fusion of features from different modalities is performed using weights:

(16) $$Fused_{1}=Concat\left(F_{\mathrm{vis}\;}⊗W_{\mathrm{vis}\;},F_{\inf\;}⊗W_{\inf\;}\right)$$

where $Fused_{1}$ denotes the features after the first fusion. Unlike the feature enhancement part, in the fusion part, where the spatial bias of the fused features towards any of the modalities results in the loss of information from the other modality, the visible features and the infrared features should have the same distribution of importance in their spatial distribution. Therefore in this paper we use the features after the first fusion to learn the spatial weight map used for fusion:

(17) M=σReLUConvConcatAvepoolFused1,MaxpoolFused1

where *M* represents the spatial weight map used for fusion. Finally, both the spatial weight map and the channel weight vectors are used to obtain the final fusion features:

(18) $$Fused=Conv\left(Concat\left(F_{\mathrm{vis}\;}⊗W_{\mathrm{vis}\;}⊛M,F_{\inf\;}⊗W_{\inf\;}⊛M\right)\right)$$

where Fused denotes the final fusion feature, the role of Conv is to reprogram the number of channels of the feature, to ensure that the fused features can be fed into the Neck section for multi-scale feature fusion.

### 3.4. Loss Function

The detection head part of the network uses a decoupled head structure with two separate branches for target classification and prediction bounding box regression respectively. The classification task uses binary cross-entropy loss (BCE Loss) and the prediction bounding box regression task uses distribution focal loss (DFL) [42] and CIoU [43].

#### 3.4.1. Binary Cross-Entropy Loss

Binary Cross-Entropy Loss (BCE Loss) is a loss function for category classification, BCE Loss is designed to ensure that the model has a low loss when the prediction is correct and a high loss when the prediction is incorrect, driving the model to optimise in the direction of correct prediction. Its mathematical expression is:

(19) $$L_{\mathrm{BCE}}=-\frac{1}{N}\sum_{i=1}^{N}\left[y_{i}\log\left(p_{i}\right)+\left(1-y_{i}\right)\log\left(1-p_{i}\right)\right]$$

where *N* is the sample size. $y_{i}$ is the true label of the i-th sample. $p_{i}$ is the probability that the model predicts this sample to be a positive class. When true label $y_{i}$ is 1, loss function focus $\log\left(p_{i}\right)$, which is the logarithm of the probability that the model predicts a positive class. When true label $y_{i}$ is 0, loss function focus $\log\left(1-p_{i}\right)$, which is the logarithm of the probability that the model predicts a negative class.

#### 3.4.2. Border Regression Loss

Targets in remote sensing images usually exist in complex scenes, resulting in ambiguity and uncertainty in the true bounding box of the target. In this paper, we use Distribution Focal Loss (DFL) [42] with CIoU [43] as the marginal regression loss. DFL makes the model focus more on samples that perform poorly on the probability distribution by taking into account the difference between the probability distribution predicted by the model and the probability distribution of the true labels. The formula for DFL is:

(20) $$L_{\mathrm{DFL}\;}\left(S_{i},S_{i+1}\right)=-\left(\left(y_{i+1}-y\right)\log\left(S_{i}\right)+\left(y-y_{i}\right)\log\left(S_{i+1}\right)\right)$$

where $S_{i}$ and $S_{i+1}$ denotes the probability of two consecutive positions predicted by the model, $y_{i}$ and $y_{i+1}$ denotes two consecutive interval points in discretised bounding box coordinates. *y* is the actual bounding box label position.

CIoU enables the target detection model to adjust the prediction frames more accurately, not only to maximise their overlap with the real frames, but also in terms of precise matching of positions and consistency of shapes, which improves the overall performance and accuracy of the detection.The CloU is:

(21) $$L_{CIoU}=1-IoU+\frac{\rho^{2}\left(b,b_{gt}\right)}{c^{2}}+\alpha v$$

where IOU is intersection of union of prediction box *b* and ground box $b_{gt}$. $\rho \left(b,b_{gt}\right)$ is European distance of the centres of *b* and $b_{gt}$. *c* is the diagonal length of the smallest closure box containing *b* and $b_{gt}$, used for normalised centre distance. *v* is used to measure the consistency of the aspect ratio, defined as $\frac{4}{\pi^{2}}{\left(\arctan\frac{w_{gt}}{h_{gt}}-\arctan\frac{w}{h}\right)}^{2}$, where w,h and $w_{gt},h_{gt}$ are the width and height of the predicted and real boxes, respectively. α is a weight parameter, is used to balance the effect of the aspect ratio, which usually depends on the value of *v*.

## 4. Experiment

To test the performance of the FAWDet proposed in this paper, we use the public datasets DroneVehicle [25] and VEDAI [26].

### 4.1. Datasets

#### 4.1.1. DroneVehicle Dataset

**Figure 5:**
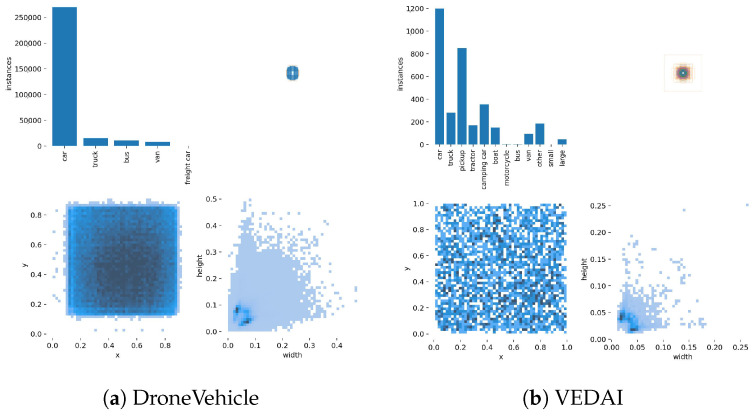
Visualisation of training data distribution for dataset DroneVehicle and dataset VEDAI.

The DroneVehicle dataset is a large-scale RGB-IR cross-modal target detection dataset captured by UAVs, which covers a wide range of scenarios ranging from daytime to nighttime, such as urban roads, residential areas, and car parks, and consists of 28,439 RGB and infrared image pairs covering the annotation of 953,087 object instances. In order to overcome, for example, the lack of performance of RGB images in low-light conditions and the noise problem in infrared images due to the lack of colour information, the DroneVehicle dataset provides an experimental basis for the study of cross-modal feature fusion, uncertainty management and target detection algorithms by providing a large number of cross-modal image pairs. Figure 5 shows information related to the labelling of objects in the DroneVehicle dataset.

#### 4.1.2. VEDAI Dataset

The VEDA dataset is designed for target recognition of small vehicles in aerial imagery and contains multi-spectral and multi-resolution images to simulate the complex environments of the real world. The VEDAI dataset consists of diverse backgrounds, such as urban roads and natural landscapes, with vehicular targets of varying directionality, and some of which suffer from occlusion and specular reflection problems, providing a rich set of challenges in the development of algorithms. The VEDA dataset is designed not only to enhance the understanding and application of small target detection techniques, but also to facilitate the advancement of related computer vision techniques in the field of aerial surveillance and reconnaissance. Figure 5 shows the information related to the labelling of objects in the VEDAI dataset.

### 4.2. Implementation Details

The neural network training process requires a large amount of arithmetic support, and in this study, we used a platform configured with an Intel Xeon-2690v4 CPU and a NVIDIA TESLA P100 GPU with 16 GB discrete video memory for the experiments. We use YOLOv8 as the main framework. The entire network is trained 200 times with weight decay set to 0.0005, momentum set to 0.937, batchsize set to 8. The training is performed using 640 resolution on the DroneVehicle dataset and 1024 resolution on the VEDAI dataset, and mosaic enhancement is used in each training session, which can greatly enrich the training data and increase the model’s ability to handle complex scenes. The experimental environment and parameter settings are shown in Table 1.

### 4.3. Evaluation Indicators

In this study we used Precision (P), Recall (R), mAP0.5, mAP0.75, and mAP0.5:0.95 to evaluate the model. P represents the proportion of true positive samples that are predicted to be positive, and a higher value of P means that the model is more accurate in the prediction of the positive class, while R represents the proportion of true positive samples that are correctly predicted to be positive, and an increase in the value of R means that the model is able to better capture the positive samples. The mAP reflects the overall accuracy of the model in multiple categories, which is one of the main evaluation indexes used in target detection tasks. mAP increase means that the model’s detection performance in each category has been improved. mAP0.5 is the mAP value when the IoU (intersection and merger ratio) threshold is set to 0.5. mAP0.5:0.95 is a more stringent index, which calculates the IoU from 0.5 to 0.95. mAP0.5:0.95 is a more stringent index, which calculates the IoU from 0.5 to 0.95. mAP0.5 is a more stringent index, which calculates the IoU from 0.5 to 0.95. mAP0.5 is a more stringent index. It calculates the average mAP in the range of IoU from 0.5 to 0.95 (with an interval of 0.05), which can more accurately evaluate the comprehensive performance of the model under different IoUs. The following formulas show how the different indicators are calculated:

(22) $$Precision\;=\frac{TP}{TP+FP}$$

(23) Recall=TPTP+FN

(24) $$AP\;=\frac{TP+TN}{TP+TN+FP+FN}$$

(25) $$mAP=\frac{1}{n}\sum_{i=0}^{n}AP_{i}=\frac{1}{n}\int_{0}^{1}P_{i}(r)dr$$

where True Positives (TP) represent the number of positive sample detection frames correctly predicted, False Positives (FP) represent the number of negative samples incorrectly predicted as positive, False Negatives (FN) represent the number of positive samples incorrectly predicted as negative and True Negatives (TN) Negatives represent number of negative samples correctly predicted to be negative.

### 4.4. Ablation Experiment

In this section, we conduct a series of ablation experiments to deeply analyse the performance of our proposed network. In this paper, YOLOv8 is used as the base network and experiments are carried out by introducing different improvements respectively, including the introduction of the cross-modal feature fusion module FFM, and the cross-modal feature enhancement module FEM. In this paper, we compare the different model configurations in terms of key performance metrics such as precision, recall, and mean average precision (mAP) for different IoU thresholds, and the analysed dataset categories are all. M1, M2, and M3 represent the dual-stream YOLOv8 using YOLOv8 to detect visible images only, using YOLOv8 to detect infrared images only, and our designed dual-stream YOLOv8, respectively. M4, and M5 are the dual-stream YOLOv8 under the improvement of applying FFM, and FEM, respectively. After the ablation study of the two datasets, the P, R, and mAP0.5, Map0.75, mAP0.5:0.95, the mean values of the five fusion metrics were quantitatively analysed. Red, blue and green colours indicate the best, second and third values, respectively. The results of one of the ablation studies on the DroneVehicle dataset and the VEDAI dataset are shown in Table 2 and Table 3.

**Figure 6:**
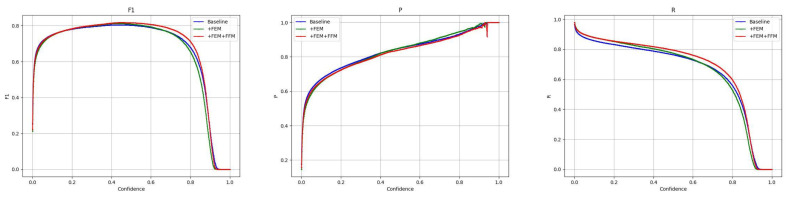
Indicators at different confidence levels on DroneVehicle.

The results of the ablation experiments on the DroneVehicle data show that the use of the dual-stream YOLOv8 architecture significantly improves the model performance compared to the single-stream models (M1 and M2), with the performance of the dual-stream YOLOv8 model (M3) on the mAP0.5 metric improving from 0.717 (M1) and 0.804 (M2) to 0.825, showing the dual-stream architecture’s ability to integrate effectiveness of visible and infrared image data. In addition, the introduction of the cross-modal feature fusion module (FFM) further improves mAP0.5 from 0.825 to 0.839 (M4), indicating that the FFM can effectively facilitate the information interaction between different modalities, thus improving the robustness of the model. The addition of the cross-modal feature enhancement module (FEM) resulted in a significant increase in the accuracy from 0.834 in M3 to 0.931 (M5), and this significant improvement proved that FEM effectively enhanced the model’s ability to recognise target details. When FFM and FEM are combined in the dual-stream YOLOv8 model (M6), while maintaining a high precision (0.840), the recall also reaches 0.796, and the best performance is achieved in the main metrics of mAP0.5, mAP0.75, and mAP0.5:0.95. The effectiveness of the cross-modal information processing module in this paper is demonstrated. In order to provide a comprehensive picture of the model’s performance, the performance of the model’s metrics at different confidence levels is shown in Figure 6.

**Figure 7:**
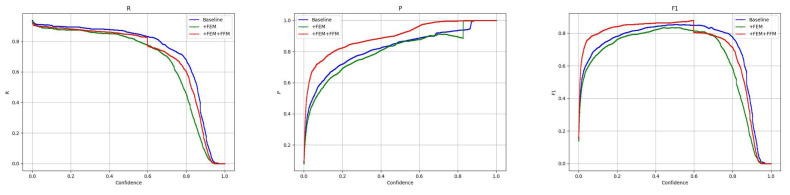
Indicators at different confidence levels on VEDAI.

Ablation experiments on VEDAI data show that the dual-stream YOLOv8 architecture (M3) significantly outperforms the single-modal configurations (M1 and M2) in terms of precision (0.798), recall (0.677), and average precision at multiple IoU thresholds (0.697 for mAP0.5 and 0.429 for mAP0.5:0.95), confirming the dual-stream architecture’s ability to fuse the visible and infrared image features with high efficiency. The dual-stream YOLOv8 (M4) with the introduction of FFM reaches 0.698 at mAP0.5 and 0.437 at mAP0.5:0.95, a significant improvement that highlights the efficacy of the FFM module in feature fusion. Meanwhile, the dual-stream YOLOv8 (M5) with integrated FEM maintains relative stability in other metrics although its mAP0.75 performance slightly decreases to 0.529, indicating the effectiveness of FEM in enhancing cross-modal feature processing capability. When both FFM and FEM are integrated into the dual-stream YOLOv8 (M6), the model performs optimally on all evaluation metrics, especially reaching the highest value of 0.439 on mAP0.5:0.95. In addition, the M6 performs well on both accuracy (0.799) and mAP0.5 (0.701). These results clearly show that the synergy of feature fusion and enhancement techniques can significantly improve the accuracy and robustness of target detection in complex multimodal scenes. In addition, we show in Figure 7 the metric transformations of this paper’s model on the VEDAI dataset at different confidence levels.

### 4.5. Comparison Experiment

In order to fully evaluate our proposed FAWDet, we conducted extensive comparative experiments on two UAV vision datasets, DroneVehicle and VEDAI.

#### 4.5.1. Comparative experiments on the DroneVehicle dataset

**Figure 8:**
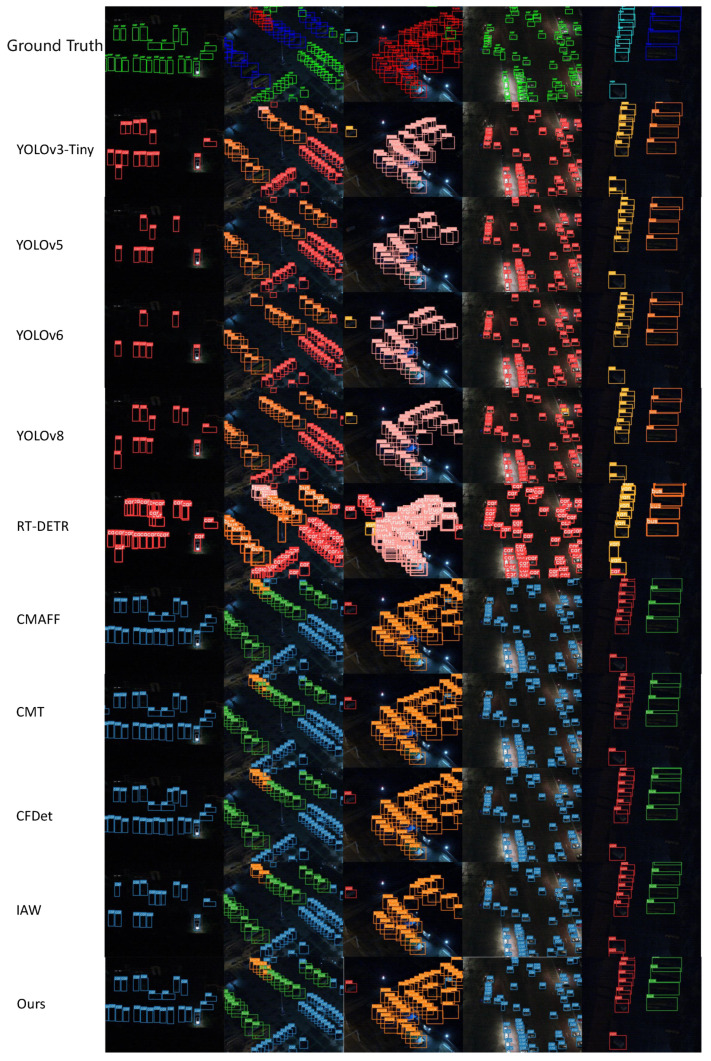
Comparison of detection results on the DroneVehicle dataset.

DroneVehicle consists of 4 main categories: Car, Truck, Bus and Van. We conducted tests on DroneVehicle according to the above environment configuration. As shown in Table 4. The experiment evaluates the performance of several target detection algorithms on the DroneVehicle UAV dataset, including single-stream models such as YOLOv3, YOLOv5, YOLOv6, and RETR, as well as a number of dual-stream multi-sensor fusion algorithms. The experiments give the metrics of mAP0.5 and mAP0.5:0.95 for different algorithms in the four categories of Car, Truck, Bus and Van as well as the overall mAP0.5 and mAP0.5:0.95, respectively. In order to show the detection effect of our method more intuitively, we chose five different scenes and compared the inference experiments on the DroneVehicle dataset using different models, and the inference results of different models are shown in Figure 8. As shown by the results in Figure 8, the method proposed in this paper has the best detection accuracy for objects in dark scenes.

Comparison experiments on the DroneVehicle dataset show that Ours achieves 0.84 on the mAP0.5 metric, outperforming other state-of-the-art single- and dual-stream models, such as YOLOv8 with a mAP0.5 of 0.804 for infrared modal image detection, and CMAFF with a mAP of 0.82 for multimodal image detection. in the mAP0.5:0.95 evaluation, the model also performs well. 0.95 evaluation, the model also performs well with up to 0.596 detection accuracy, which is significantly higher than other detection models, a result that validates the advantages of our model in terms of accuracy and robustness. In terms of vehicle category-specific performance, especially in the “Truck” and “Van” categories, our model achieves a mAP0.5 of 0.792 and 0.643, respectively, which further highlights its superior performance in handling vehicles of different sizes and types. vehicles of different sizes and types. Furthermore, despite reaching 53 in terms of frame rate (FPS), slightly lower than the fastest model, YOLOv3-Tiny, this frame rate ensures a good balance between real-time performance and accuracy, given the high accuracy of the model and the demands of complex data processing. Our proposed YOLOv8 two-stream model, by integrating the Cross-modal Feature Fusion Module (FFM) and Cross-modal Feature Enhancement Module (FEM), achieves the optimal performance in all categories, and also confirms its high efficiency and applicability in real-world applications.

**Figure 9:**
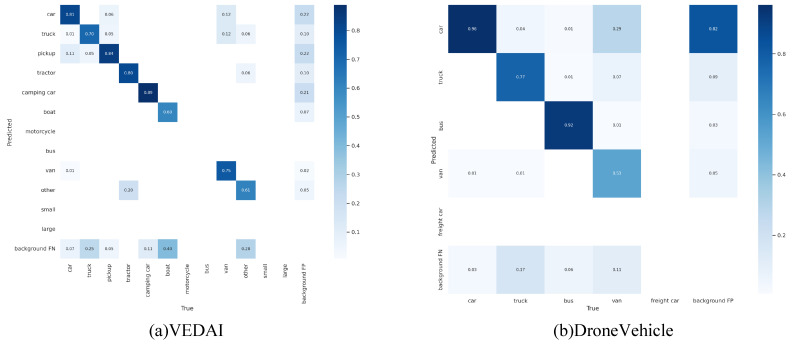
Confusion matrix generated by the model in this paper on different datasets.

In order to visualise the performance of the model in this paper, the confusion matrices generated by the model in this paper on the two datasets are presented in Figure 9. The horizontal coordinates in Figure 9 indicate the true category of each labelled box, the vertical coordinates indicate the categories predicted by this paper’s method for each category, and the data in the squares indicate the probability of occurrence of different combinations. The data show that the method in this paper correctly classifies each category with a low false alarm rate and high stability.

#### 4.5.2. Comparative Experiments on the VEDAI Dataset

**Figure 10:**
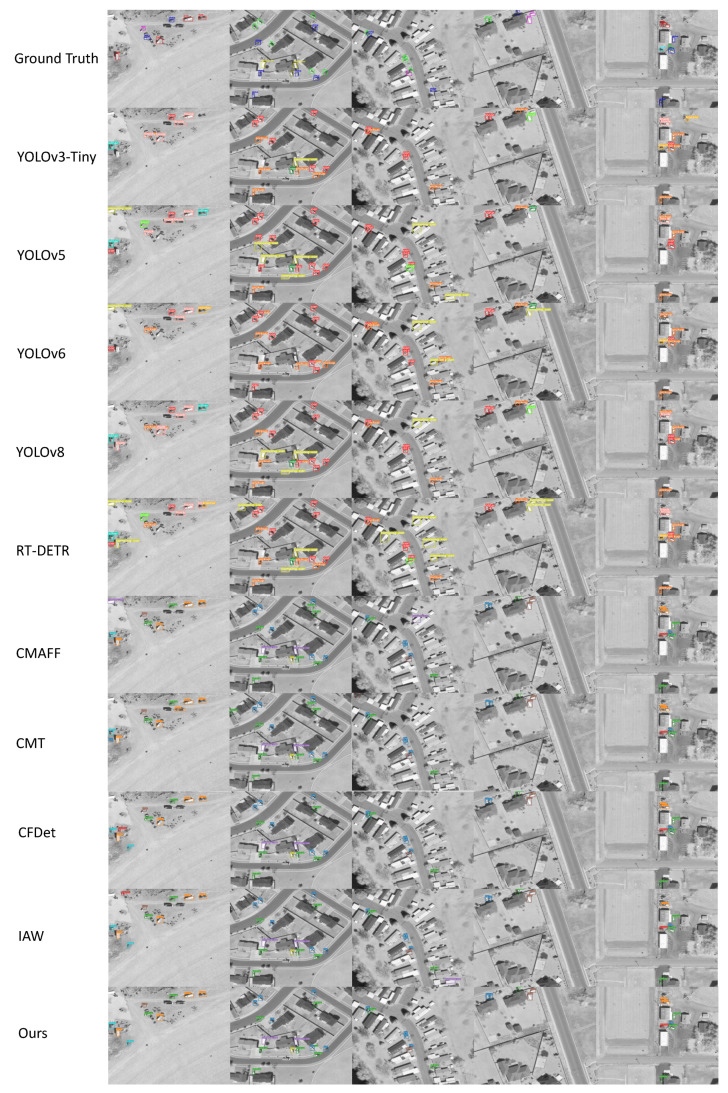
Comparison of detection results on the VEDAI dataset.

In the VEDAI dataset we selected 8 major categories for comparison, such as Car, Truck, Boat, etc., and we also tested them on the VEDAI dataset according to the above comparison model. The results of the comparison experiment on VEDAI dataset are shown in Table 5. The experiment evaluates the performance of the same batch of algorithms on the VEDAI UAV vision dataset. In order to show more intuitively the detection effect of our method on the VEDAI dataset, we still choose five different scenarios for inference experiment comparison, the inference results of different models are shown in Figure 10, and the method proposed in this paper can still get satisfactory results in the task of target detection in complex environments.

Comparison experiments on the VEDAI dataset show that our model achieves 0.692 on the mAP0.5 metric, outperforming the CMAFF model’s 0.68 and the CMT model’s 0.679. On the mAP0.5:0.95 metric, our model leads the CMAFF’s 0.426 and the CMT’s 0.409 with a score of 0.437, demonstrating its stability and robustness in high-precision target detection. The gap between the single-stream and dual-stream models on VEDAI is somewhat smaller than that of DroneVehicle, but our designed model still achieves the best performance on most categories. In terms of category-specific detection capability, our model achieves mAP0.5 of 0.593, 0.91 and 0.931 on Truck, Pickup and Tractor categories, respectively, which highlights its strong ability to accurately identify different vehicle types. Although in terms of processing speed, our model is slightly lower than some single-stream models at a rate of 51 frames per second, this frame rate still represents a practical balance between performance and real-time performance considering the complexity of the data it processes and the high accuracy required. The confusion matrix in Figure 9 confirms the stability of the method in this paper on VEDAI.

## 5. Conclusions

In this paper, a dual-stream real-time detector is proposed, which is capable of utilising visible and infrared images simultaneously, effectively improving the stable detection performance in extreme environments such as night and fog. In order to counteract the inevitable information loss during network deepening, this paper designs a cross-modal feature enhancement module (FEM), which significantly improves the detection of weak targets by enhancing the differential features between different modalities. Aiming at the differences of different modal features, this paper further designs the cross-modal feature fusion module (FFM) with a three-stage fusion strategy to optimise the feature fusion from three dimensions: spatial, channel and overall. Through extensive experiments on two datasets, the method in this paper proves to achieve state-of-the-art performance in detecting weak targets. The designed dual-stream detector embedding FEM as well as FFM in this paper has significant advantages in enhancing the detection performance in extreme environments, and FEM and FFM can be widely applied to dual-stream feature extraction base detectors.

## Figures and Tables

**Table 1:** Environment and parameterisation of the experiment.

Parameters	Configuration
CPU	Intel Xeon-2690v4
GPU	NVIDIA TESLA P100 16 GB
System	Ubuntu 18.04
Deep learning architecture	Pytorch1.9.2 + Cuda11.4 + cudnn11.4
Training Epochs	200
Batch size	8
Weight Decay	0.0005
Momentum	0.937

**Table 2:** Ablation findings on the DroneVehicle dataset.

Model	M1 (Vis)	M2 (Inf)	M3	M4	M5	M6
FFM	×	×	×	✓	×	✓
FEM	×	×	×	×	✓	✓
Precision	0.757	0.796	0.834	0.835	0.931	0.840
Recall	0.676	0.762	0.781	0.795	0.808	0.796
mAP0.5	0.717	0.804	0.825	0.839	0.838	0.84
mAP0.75	-	-	0.701	0.712	0.716	0.718
mAP0.5:0.95	0.433	0.576	0.585	0.591	0.596	0.596

**Table 3:** Ablation findings on the VEDAI dataset.

Model	M1 (Vis)	M2 (Inf)	M3	M4	M5	M6
FFM	×	×	×	✓	×	✓
FEM	×	×	×	×	✓	✓
Precision	0.464	0.549	0.798	0.721	0.687	0.799
Recall	0.533	0.496	0.677	0.628	0.639	0.671
mAP0.5	0.521	0.569	0.697	0.698	0.674	0.701
mAP0.75	-	-	0.522	0.549	0.542	0.529
mAP0.5:0.95	0.310	0.353	0.429	0.437	0.425	0.439

**Table 4:** Comparison experiments on DroneVehicle.

Method	Modality	Car	Truck	Bus	Van	mAP0.5	mAP0.5:0.95	FPS
YOLOv3-Tiny [16]	Visible	0.850	0.507	0.833	0.351	0.635	0.352	**166**
YOLOv5 [34]	Visible	0.878	0.503	0.827	0.401	0.652	0.377	75
YOLOv6 [44]	Visible	0.883	0.509	0.837	0.354	0.646	0.378	84
YOLOv8 ^1^	Visible	0.901	0.602	0.881	0.483	0.717	0.433	89
RT-DETR [45]	Visible	0.84	0.37	0.778	0.198	0.546	0.295	32
YOLOv3-Tiny [16]	Thermal	0.956	0.67	0.907	0.0489	0.755	0.519	166
YOLOv5 [34]	Thermal	0.968	0.673	0.901	0.534	0.769	0.533	75
YOLOv6 [44]	Thermal	0.967	0.658	0.899	0.45	0.743	0.518	84
YOLOv8 ^1^	Thermal	0.973	0.738	0.919	0.584	0.804	0.576	89
RT-DETR [45]	Thermal	0.951	0.593	0.873	0.327	0.686	0.47	32
CMAFF [46]	Visible + Thermal	0.975	0.762	0.941	0.604	0.82	0.576	43
CMT [38]	Visible + Thermal	0.976	0.768	0.939	0.606	0.822	0.576	25
IAW [47]	Visible + Thermal	0.898	0.625	0.892	0.487	0.726	0.416	50
CFDet [21]	Visible + Thermal	0.976	0.774	0.94	0.632	0.83	0.582	51
Ours	Visible + Thermal	**0.977**	**0.792**	**0.946**	**0.643**	**0.84**	**0.596**	53

^1^ https://github.com/ultralytics/ultralytics (accessed on 14 June 2024).

**Table 5:** Comparison experiments on VEDAI.

Method	Modality	Car	Truck	Pickup	Tractor	Camping-Car	Boat	Van	Other	mAP0.5	mAP0.5:0.95	FPS
YOLOv3-Tiny [16]	Visible	0.847	0.501	0.73	0.692	0.805	0.454	0.513	0.543	0.565	0.297	169
YOLOv5 [34]	Visible	0.761	0.308	0.563	0.43	0.699	0.17	0.489	0.443	0.428	0.25	78
YOLOv6 [44]	Visible	0.663	0.221	0.504	0.266	0.539	0.378	0.337	0.33	0.36	0.214	86
YOLOv8 ^1^	Visible	0.824	0.406	0.706	0.669	0.745	0.419	0.441	0.481	0.521	0.31	117
RT-DETR [45]	Visible	0.787	0.462	0.828	0.88	0.781	0.493	0.628	0.559	0.602	0.392	31
YOLOv3-Tiny [16]	Thermal	0.823	0.218	0.698	0.563	0.62	0.323	0.541	0.301	0.454	0.261	169
YOLOv5 [34]	Thermal	0.789	0.321	0.73	0.502	0.626	0.397	0.505	0.398	0.474	0.271	78
YOLOv6 [44]	Thermal	0.786	0.296	0.733	0.587	0.717	0.149	0.348	0.407	0.447	0.263	86
YOLOv8 ^1^	Thermal	0.876	0.546	0.852	0.779	0.696	0.456	0.54	0.371	0.569	0.353	117
RT-DETR [45]	Thermal	0.798	0.502	0.688	0.575	0.687	0.404	0.697	0.249	0.52	0.327	31
CMAFF [46]	Visible + Thermal	**0.917**	0.566	0.908	0.887	0.895	0.591	**0.793**	0.564	0.68	0.426	41
CMT [38]	Visible + Thermal	0.902	0.586	0.857	**0.962**	0.889	0.594	0.764	0.556	0.679	0.409	25
IAW [47]	Visible + Thermal	0.92	0.622	0.917	0.843	0.882	0.653	0.729	0.597	0.685	0.422	45
CFDet [21]	Visible + Thermal	0.908	0.575	0.853	0.96	0.835	0.68	0.747	0.61	0.685	0.428	47
Ours	Visible + Thermal	0.900	**0.593**	**0.91**	0.931	**0.907**	**0.637**	0.787	**0.565**	**0.692**	**0.437**	51

^1^ https://github.com/ultralytics/ultralytics (accessed on 14 June 2024).

## Data Availability

Data from this study are available upon reasonable request from the corresponding authors.
